# The Relationship between Mental and Physical Minor Health Complaints and the Intake of Dietary Nutrients

**DOI:** 10.3390/nu15040865

**Published:** 2023-02-08

**Authors:** Hiroyo Kagami-Katsuyama, Maremi Sato-Ueshima, Kouji Satoh, Yuko Tousen, Hidemi Takimoto, Mari Maeda-Yamamoto, Jun Nishihira

**Affiliations:** 1 Department of Medical Management and Informatics, Hokkaido Information University, Ebetsu 069-8585, Japan; 2 Department of Nutritional Epidemiology and Shokuiku, National Institutes of Biomedical Innovation, Health and Nutrition, Shinjuku-ku 162-8636, Japan; 3 Institute of Food Research, National Agriculture and Food Research Organization, Tsukuba 305-8643, Japan

**Keywords:** minor health complaints (MHCs), psychosomatic disorder, presenteeism, dietary intakes of nutrients

## Abstract

Presenteeism is a problem that needs to be solved urgently, both for individual workers and for society overall. In this report, we propose the concept of MHC, which refers to mild mental and physical complaints subjectively perceived by individuals that are not caused by illness. We also planned to examine what kind of physical and mental disorder MHC is and whether food is effective as a method of self-care for MHC. First, we conducted “the comprehensive survey to establish an integrated database of food, gut microbiome, and health information” (the “*Sukoyaka* Health Survey”) and obtained data on psychosomatic disorders and intakes of dietary nutrients. As a result, through factor analysis and item response theory analysis, we found the following specific examples of MHC: lack of vigor, irritability, fatigue, and somatic complaints. In addition, analysis of the relationship between these four MHC levels and the intake dietary nutrients indicated that they are closely related and that MHC levels can be improved by consuming sufficient amounts of multiple nutrients.

## 1. Introduction

Recently, the problems of lowered work productivity (presenteeism) and economic loss have emerged, caused by mental and physical disorders such as sleep debts and excessive stress. Economic losses due to presenteeism in Japan as a whole have been calculated by the WHO-HPQ, a presenteeism scale, to be JPY 19.3 trillion per year, with the average annual loss per worker amounting to several hundred thousand yen [1]. On the other hand, it has become evident that workers with a high level of well-being without psychosomatic disorders have a high level of productivity and creativity [2]. Therefore, improving individual productivity, reducing economic loss, improving mental and physical disorders, and forming a healthy, long-lived, and energetic society is an urgent problem.

Given this situation, we first considered that there are two types of disorders that cause presenteeism: psychosomatic disorders caused by illness and minor psychosomatic disorders not caused by illness. Thus, in this report, we refer to psychosomatic disorders that are not related to illness as minor health complaints (MHCs), which are subjectively felt by the individual. It is not hard to imagine that MHCs can result in lower productivity; therefore, it is important to find ways to improve MHCs not only for an individual but for society as a whole.

MHCs are not a disease; thus, many people hope to improve their complaints through self-care. Self-care can be conducted in several ways such as diet changes, exercise, bathing, and moderate sun exposure. In particular, it has long been empirically accepted that eating and health are closely related. For example, there are some phrases that indicate or partially indicate this, such as “An apple a day keeps the doctor away” (an English proverb), “Drink morning tea even if you come back from far away”, and “A persimmon turns red, a doctor turns blue” (proverbs in Japanese). People today strongly view food as an important way to maintain good health. The concept of functional foods has taken root in various countries, including Japan, and each country has established systems around functional foods [3]. Thus, according to reports by international research organizations, the functional food market continues to grow consistently [4].

Furthermore, there are many reports on how daily diet relates to health in people with illnesses [5,6] as well as randomized controlled trial studies examining how functional foods relate to health in healthy people with minor mental and physical complaints [7,8]. On the other hand, there have not been many studies on the relationship between daily diet and health among healthy people with mild physical and mental disorders. In this study, we examined whether MHCs can be improved through daily diet and micronutrient intake changes.

## 2. Materials and Methods

### 2.1. Study Design and Population

We performed this study using data obtained from “the comprehensive survey to establish an integrated database of food, gut microbiome, and health information” (the “*Sukoyaka* Health Survey”). The “*Sukoyaka* Health Survey” was conducted on Japanese men and women aged 20 years or older and younger than 80 years except for “a patient with serious cerebrovascular disease, heart disease, liver disease, kidney disease, gastrointestinal disease, or infection requiring notification”. (COVID-19 infection was included in the list of infections requiring notification.) In the “*Sukoyaka* Health Survey”, we conducted two surveys on the same subjects: one in summer and one in winter. The “*Sukoyaka* Health Survey” was conducted in fiscal 2019 and 2020 as part of the Strategic Innovation Creation Program (SIP) project. In this study, we analyzed data obtained from subjects who gave written informed consent in fiscal years 2019 and 2020 at Hokkaido Information University.

### 2.2. Measurement of the Level of Psychosomatic Disorders

The “Brief Job Stress Questionnaire (BJSQ)” is a 29-item questionnaire querying psychological and physical stress reactions. The group prepared this questionnaire through the Stress Measurement Study Group of the “Study Group on the Prevention of Work-related Diseases” of the Ministry of Health, Labour, and Welfare [9].

### 2.3. Estimation of Dietary Nutrient Intakes (Nutritional Survey)

Subjects completed a food record questionnaire (breakfast, lunch, dinner, and snack) on any given day, weighing themselves on a kitchen scale as appropriate. Additionally, we took dietary photographs whenever possible. The supervising dietitian checked the content of the food record questionnaire and the dietary photographs, asked the subjects about insufficient information in calculating nutrition, and calculated the daily nutritional intake from the food record questionnaire. Calculations followed the Japanese Food Standard Component Table 2015 (Seventh Revised Edition) [10].

### 2.4. Analysis of the Relationship between the MHC Levels and the Intake of Dietary Nutrients

We analyzed the relationship between the MHC levels (lack of vigor, irritability, fatigue, and somatic symptoms) and dietary nutrient intake calculated from the Nutritional Survey. The dietary nutrient intakes were compared between relatively high-MHC-level subjects and relatively low-level subjects. Relatively higher and lower MHCs were above and below the median for each complaint (lack of vigor, irritability, fatigue, and somatic symptoms).

### 2.5. Statistical Analysis

We performed exploratory factor analysis (EFA) and confirmatory factor analysis (CFA) using R software packages [11,12,13]. In addition, the response properties of stress response measures were calculated using item response theory (IRT) [14,15]. Unidimensional structures of each item were confirmed, and two-parameter logistic (2PL) IRT analysis was performed.

As for EFA, CFA, and IRT, the answers to the BJSQ were converted to 0-0-1-1 in this order and in four steps of 1-2-3-4. The exceptions were the answers to Q01 (I have been very active), Q02 (I have been full of energy), and Q03 (I have been lively). We set 1-2-3-4 as 1-1-0-0 and unified the orientation of the point. Thus, the points of the negative answers tended relatively higher in all questions. The response curves were demonstrated by the use of averaged numbers of mass of each item. Except for the EFA, CFA, and IRT, the scoring for each question on the BJSQ followed that previously reported [9].

Mann–Whitney U tests were used for intergroup comparisons. A *p*-value of <0.05 was considered significant. For analysis, SPSS ver. 25 (IBM Japan, Tokyo, Japan) was used.

### 2.6. Ethics

The “*Sukoyaka* Health Survey” was conducted following the ethical principles based on the Declaration of Helsinki (revised by the World Medical Association Fortareza General Assembly in October 2013) and in compliance with the Ethical Guidelines for Medical Research for Persons (revised by the Ministry of Education, Culture, Sports, Science and Technology and the Ministry of Health, Labour, and Welfare on 28 February 2017). We obtained written informed consent from all subjects. The Bioethics Committee of Hokkaido Information University reviewed and approved the feasibility of clinical trials and the ethical and scientific validity (approval date: 22 April 2019; approval number: 2019-04).

## 3. Results

### 3.1. Baseline Characteristics

**Figure 1:**
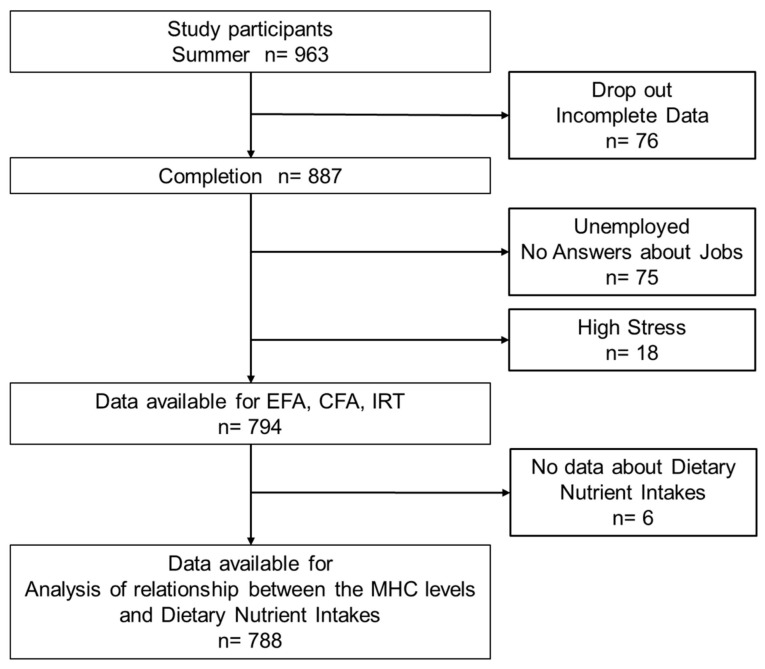
Flowchart of subject selection and participation.

Figure 1 shows a detailed flowchart of the population selected for this study. First, according to the questionnaire’s response, 887 subjects who completed this study were classified as occupational and unemployed. Moreover, we excluded 75 subjects who answered “no occupation or no answer” from all the analyses. In addition, 18 subjects with high psychological stress reactions throughout the year were excluded based on the “Manual for Implementation of Stress-Check System” [9]. Ultimately, we included 794 participants in the analysis as the “working subject population”.

Table 1 shows demographic information of the analyzed population (794 persons: 2019 fiscal year: 520 persons; 2020 fiscal year: 274 persons). The gender composition was 3:7 for male-to-female participants. The majority of the age group was from 40 to 60 years old.

### 3.2. Characterization of the Analysis Population Using the BJSQ

**Figure 2:**
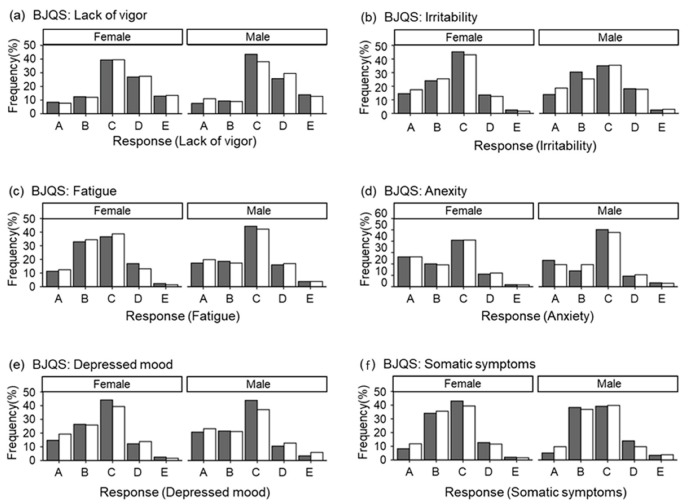
The Brief Job Stress Questionnaire. Gray: summer; white: winter. (**a**) Responses (lack of vigor), (**b**) responses (irritability), (**c**) responses (fatigue), (**d**) responses (anxiety), (**e**) responses (depressed mood), and (**f**) responses (somatic symptoms). *x*-axis—A: mild, B: slightly mild, C: normal, D: slightly severe, E: severe. *y*-axis—frequency of subjects (%).

Figure 2 presents the results of six psychosomatic responses (lack of vigor, irritability, fatigue, anxiety, depressed mood, and somatic symptoms) caused by stress assessed by the BJSQ. The method for determining the degree of psychosomatic disorders followed the “Manual for Implementation of Stress-Check System” of the Ministry of Health, Labour, and Welfare [9]. Consequently, comparisons between summer (gray) and winter (white) showed no inter-seasonal differences in the distributed frequencies of subjects, both in males and females, in all psychosomatic responses. In the subsequent analysis, we describe notable results of the combined analysis of summer and winter data.

First, about 40% of the subjects were judged “C: normal” in all six psychosomatic responses. Regarding lack of vigor, approximately 20% of subjects in both sexes were confirmed to have decreased vigor (“A: mild, B: slightly mild”) (Figure 2a). Irritability and fatigue were observed in approximately 15% of subjects, with “D: slightly severe” and “E: severe” in approximately 3% (Figure 2b,c); anxiety and depressed mood were observed in approximately 10% of subjects, with “D: slightly severe” and “E: severe” in approximately 3% (Figure 2d,e). Finally, for somatic symptoms, unlike the other five items, both men and women showed about 80% of the total in both “B: slightly mild, C: normal” (Figure 2f). Additionally, “D: slightly severe, E: severe” was combined, totaling approximately 13%.

Next, we conducted an EFA on summer data using the 29 items of the BJSQ psychological and physical stress reactions in the “working subject population”. First, we examined the sample validity of Kaiser–Meyer–Olkin to confirm that factorial analyses were feasible with the present data. Given the KMO level of 0.86, we confirmed that the sample size could be satisfactorily subjected to factorial analyses. When we then examined the optimal number of factors, a five-factor solution was concluded to be valid from a screen plot showing the decay situation of eigenvalues. A five-factor solution was also supported in parallel analyses and the MAP criteria.

We adopted a five-factor solution (ML1–ML5) from the above findings. Then, the EFA method and oblique rotation method (Oblimin rotation method) were carried out and analyzed using the maximum likelihood approach. Table 2 shows the oblique solution pattern matrix of the factor analysis. Compared with the published report (x), ML1 consisted of the two published scales (anxiety and depressed mood), and two questions were from one published scale (somatic symptoms). ML2 consisted of one published scale (lack of vigor), ML3 consisted of one published scale (irritability), ML4 consisted of one published scale (fatigue), and ML5 consisted of the questions excluding the two questions classified as ML1 from the one published scale (somatic symptoms).

Next, we performed a CFA on the winter data. This confirmed the validity of the above five scales: ML1 consisted mainly of items about anxiety and depressed mood; ML2 consisted of items about lack of vigor; ML3 consisted of items about irritability; ML4 consisted of items about fatigue; and ML5 consisted of items about somatic symptoms. As for assessing the goodness-of-fit, the values of the comparative fit index (CFI), Tucker–Lewis index (TLI), and standardized root mean square residual (SRMR) were 0.889, 0.878, and 0.056, respectively; accordingly, we concluded that the assumed five-factor scale was valid.

### 3.3. Evaluation of MHCs Using the BJSQ

**Figure 3:**
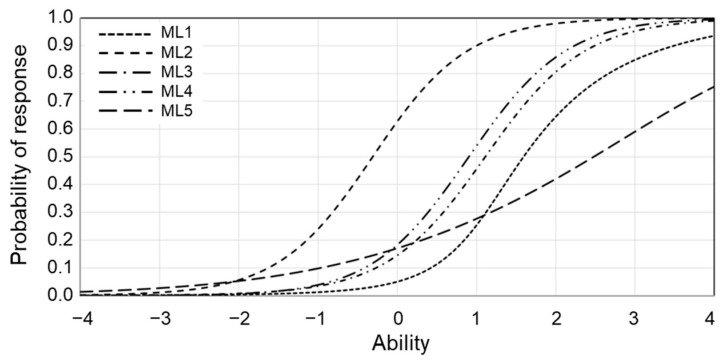
Item characteristic curve for psychosomatic disorder level. *x*-axis—ability (psychosomatic disorder level). *y*-axis—probability of response.

In addition, we attempted to distinguish relatively mild psychosomatic disorders from less mild psychosomatic disorders. Based on the factor analysis results, we judged there to be uni-dimensionality in all the items and used the five factors derived in this study. Figure 3 shows the average for each summary of each factor, and item–response curves are shown. There were five factors: ML1–ML5. The *x*-axis reflects the psychosomatic disorder level (θ) as a latent trait. The findings showed that the ML1–ML4 had high slopes and discriminatory power.

On the other hand, the slope of the ML5 was very gradual, confirming that it appeared at a broad psychosomatic disorder level. The psychosomatic disorder level shown here is the threshold. This property indicates the projected point to the *x*-axis at the corresponding response rate of 0.5. Looking at each threshold of the five factors, the lack of vigor appeared in the early stage, with the highest threshold on the left. Next, fatigue, irritability, and finally anxiety and depression mood followed.

Accordingly, we defined lack of vigor, irritability, fatigue, and somatic symptoms, which appeared in the stage of the mild psychosomatic disorders as MHC. In subsequent analyses, the degree of each MHC was quantified by reference to Table 2: lack of vigor in Q1–Q3, irritability in Q4–Q6, fatigue in Q7–Q9, and somatic symptoms in Q19–26 and 28.

### 3.4. Relationship between MHC and the Intake of Dietary Nutrients

We investigated the relationship between four items of MHC and thirty-nine items pertaining to the intake of dietary nutrients (sodium, potassium, calcium, magnesium, phosphorus, iron, zinc, copper, vitamin A, retinol, beta-cryptoxanthin, beta-carotene equivalents, vitamin D, vitamin E, vitamin K, vitamin B1, vitamin B2, niacin, vitamin B6, vitamin B12, folic acid, pantothenic acid, vitamin C, saturated fatty acids, monounsaturated fatty acids, polyunsaturated fatty acids, cholesterol, total dietary fiber, soluble dietary fiber, insoluble dietary fiber, n-3 fatty acids, n-6 fatty acids, triacylglycerol equivalents, manganese, iodine, selenium, chromium, molybdenum, and biotin). First, as for lack of vigor, irritability, fatigue, and somatic symptoms, we divided the population into a subpopulation below the median and a subpopulation above the median based on MHC level. (For the median, anxiety was 7 points, irritability was 6 points, fatigue was 6 points, and somatic symptoms was 15 points.) Then, as for lack of vigor, irritability, fatigue, and somatic symptoms, we conducted inter-subpopulation comparisons and confirmed whether significant differences were present or not. Table 3 shows seventeen nutrients with significant differences shared by three or more items of MHCs. We also evaluated the relationship between caloric intake and MHC, but no significant relationship was found.

**Figure 4:**
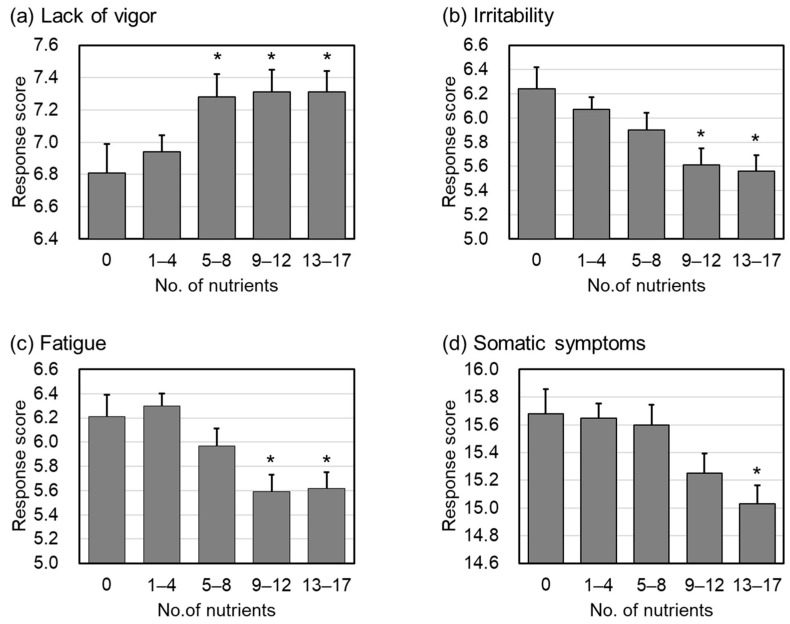
The mean MHC levels in each subgroup. (**a**) Response score (lack of vigor), (**b**) response score (irritability), (**c**) response score (fatigue), (**d**) response score (somatic symptoms). Bar charts are shown as the mean ± standard error. Asterisks show significant difference compared with the 0N group. As for the lack of vigor, a higher response score indicates better psychosomatic status, and a lower response score indicates better status for irritability, fatigue, and somatic complaints.

Next, we set the cut-off values for these seventeen nutrients. The cut-offs were set so that all four items of MHC were above “the low subpopulation” in Table 3 and below “the high subpopulation” in Table 3. For each subject, we counted how many of the seventeen nutrients shown in Table 3 were at or above the cut-off. First, we classified subjects according to the number (0/1–4/5–8/9–12/13–17) of nutrient intake levels above the cut-offs. Hereafter, we refer to the subgroups as the 0N group, 1–4N group, 5–8N group, 9–12N group, and 13–17N group, respectively. Figure 4 shows the mean MHC levels for each classified group. For every four items of MHC, we examined whether there were significant differences between the 1–4N group, 5–8N group, 9–12N group, and 13–17N groups, using the 0N groups as a control. The findings showed significant differences between the 5–8N, 9–12N, and 13–17N groups in lack of vigor; between the 9–12N and 13–17N groups in irritability; between the 9–12N and 13–17N groups in fatigue; and within the 13–17N group in somatic symptoms.

## 4. Discussion

Tosen et al. reported that stress, sleep quality, and comprehensive health questionnaire items are required to assess mild psychosomatic disorder [16]. Using the “Brief Job Stress Questionnaire” (BJSQ), which is also presented in Tosen’s report [16], this study examined minor health complaints (MHCs). The BJSQ can be used to measure and evaluate psychosomatic disorder conveniently; in addition, it is a questionnaire with high validity [9]. First, we characterized the present population using the BJSQ. In Figure 2, the subject frequencies of the six psychosomatic responses are shown as: (a) lack of vigor, (b) irritability, (c) fatigue, (d) anxiety, (e) depressed mood, and (f) somatic symptoms. Approximately 40% of the subjects were judged normal (C) in all six psychosomatic responses, showing a similar tendency to that reported previously [17].

On the other hand, the frequency of subjects with a lack of vigor, irritability, and fatigue was approximately 5–7% lower than that reported previously, and the frequency of subjects with high anxiety and depressed mood was approximately half of that reported previously [17]. In addition, the frequency of subjects with high somatic symptoms was 6–9% lower in females and 2–4% lower in males compared with that previously reported [17]. The distribution frequency of “psychosomatic disorder “ in this working subject population compared with that reported previously [17] shows that the population was slightly less stressed. Therefore, we decided to use factor analyses to investigate whether such populations could be used as controls to capture psychosomatic disorders on a scale similar to that previously reported. Thus, we conducted factor analysis to examine whether it is reasonable to classify psychosomatic disorder into six categories even in the group with relatively mild psychosomatic disorders.

The results showed that the classification performed in this report using factor analysis had two minor points that differed from the classification method for psychosomatic disorders used previously [9,17]. First, the anxiety and depressed moods, which had previously been evaluated as separate reaction scores, were separated into a single group, anxiety + depression. In the second point, two questions, Q27 (no appetite) and Q29 (not sleeping well), which were initially grouped into somatic complaints, were integrated into the same group with a new ML1 (anxiety + depressed mood). Therefore, somatic complaints (physical complaints) were reduced to nine questions (ML5) (Table 2). In addition, ML2, ML3, and ML4 could be separated into three: lack of vigor, irritability, and fatigue, as in the previous report [9,17].

Despite these slight differences, we can conclude that the results of the present factor analysis are generally consistent with the scales presented in previous reports [9,17]. In subsequent analyses, however, we adopted ML1–ML5 as a classification of psychosomatic disorders that would be more suited to the present population.

Then, IRT was employed to analyze what kind of psychosomatic disorders appeared according to the degree of psychosomatic disorder. Figure 3 shows the item–response curves for each factor scale. To restate, there were five factors used here: ML1, consisting mainly of items about anxiety and depressed mood; ML2, consisting of items about lack of vigor; ML3, consisting of items about irritability; ML4, consisting of items about fatigue; and ML5, consisting of items about somatic symptoms. Examining the respective thresholds, among the five factors, “lack of vigor” exhibited the leftmost threshold and emerged in the early psychosomatic disorder level. Next, “fatigue” and “irritability” appeared, and “anxiety and depressed mood” appears the latest. “Lack of vigor” also emerged at lower psychosomatic disorder levels, indicating that a “lack of vigor” is already present when other factors reach the threshold. The results suggest that “lack of vigor” is a complaint recognized at a relatively low level of psychosomatic disorder, followed by “irritability” and “fatigue” and finally “anxiety and depression” at the highest level of psychosomatic disorder. The slope for “somatic complaints” was very gradual, indicating that they appeared at a wide range of levels of psychosomatic disorder. These results were consistent with those in previous reports [9].

Previous reports have noted that the most notable symptom to look for when observing more serious stress problems is depression and that appropriately addressing anxiety and depression is extremely important in mental health practice [9]. Therefore, in this study, with the exception of these complaints, we defined MHCs as a lack of vigor, irritability, fatigue, and somatic symptoms that may appear in the relatively mild stage of psychosomatic disorders. The degree of MHC was measured by Q1–Q3, Q4–Q6, Q7–Q9, and Q19–26, 27, and 29, as shown in Table 2, for lack of vigor, irritability, fatigue, and somatic symptoms. The degree of MHC could be measured by Q1–Q3; irritability could be measured by Q4–Q6; fatigue could be measured by Q7–Q9; and somatic symptoms could be measured by Q19–26, 27, and 29, as shown in Table 2.

The relationships between individual scores on the four MHC items and nutrient intakes from the diet were examined. The results showed that the 17 nutrients listed in Table 3 were associated with almost all four MHC items. Furthermore, as shown in Figure 4, for each of these 17 components, a relatively high intake of multiple components was associated with improvement in the four MHC items.

The following is a brief summary and discussion of the 17 nutrients of note that may be related to MHCs. First, folic acid has been reported to improve depressive symptoms [18]. Although depressive symptoms are not part of MHCs, folic acid may also have effects on MHC with milder levels of psychosomatic disorders.

In addition, dietary fiber, especially insoluble fiber, improves the intestinal environment [19], and improvement of the intestinal environment has been reported to lead to improved health [20]. Dietary fiber may also improve defecation [21]; some reports suggest that improved defecation may lead to an improved quality of life [22,23].

It is understood that minerals and vitamins are necessary for maintaining a healthy mind and body; however, it is interesting that vitamin B6, which promotes the biosynthesis of neurotransmitters (dopamine, serotonin, GABA, etc.) [24], and carotene, which is reported to relieve mental stress [25], were among the 17 nutrients in this study.

Finally, although we have shown that MHC and dietary nutrient intake are closely related, the results of the analyses in this report cannot demonstrate a causal relationship. However, as noted above, some of the nutrients that may be important in improving MHC in this study have already been reported to contribute directly or indirectly to improving physical and mental health problems. Therefore, it is reasonable to assume that nutrient intakes affect MHC levels. Notably, this report suggests that taking relatively high amounts of several nutrients may be more conducive to improving MHC levels. This would imply that daily dietary nutrients are crucial for preventing MHCs in the healthy individuals included in this analysis.

However, a limitation of this study is that we did not consider the different nutrient intake requirements of different individuals. Several nutrients in this study did not have appropriate recommended intakes in the general standards of Japan. In addition, a detailed assessment of physical activity and expiratory gas analysis is mandatory to calculate calorie requirements; however, we did not include these two factors in the study. Thus, it is advisable to assess that nutrient intake requirements differ between individuals with different levels of physical activity and body size. Based on these facts, it will be necessary to make an overall judgment about how the amount of nutrients we should take to improve MHC levels by referring to the threshold levels established in this study and the generally recommended intake.

## 5. Conclusions

In this report, we proposed the idea of “minor health complaints” (MHCs), which refers to psychosomatic disorders that are not related to illness and are subjectively felt by the individual. Specifically, we treated four physical and mental complaints (decreased vitality, irritability, fatigue, and somatic complaints) as MHCs. Furthermore, we have shown that MHC and dietary nutrient intake are closely related, and taking relatively high amounts of several nutrients may be more conducive to improving MHC levels.

## Figures and Tables

**Table 1:** Characteristics of participants in this study.

Fiscal Year	Age Groups	Total		Male		Female	
		*n*	%	*n*	%	*n*	%
All	20 to 29	42	5.3	13	5.5	29	5.2
	30 to 39	118	14.9	40	16.8	78	14.0
	40 to 49	232	29.2	70	29.4	162	29.1
	50 to 59	262	33.0	66	27.7	196	35.3
	60 to 69	116	14.6	40	16.8	76	13.7
	70 and more	24	3.0	9	3.8	15	2.7
	all	794	-	238	-	556	-
2019	20 to 29	21	4.0	8	4.9	13	3.6
	30 to 39	73	14.0	27	16.6	46	12.9
	40 to 49	148	28.5	45	27.6	103	28.9
	50 to 59	170	32.7	44	27.0	126	35.3
	60 to 69	86	16.5	30	18.4	56	15.7
	70 and more	22	4.2	9	5.5	13	3.6
	all	520	-	163	-	357	-
2020	20 to 29	21	7.7	5	6.7	16	8.0
	30 to 39	45	16.4	13	17.3	32	16.1
	40 to 49	84	30.7	25	33.3	59	29.6
	50 to 59	92	33.6	22	29.3	70	35.2
	60 to 69	30	10.9	10	13.3	20	10.1
	70 and more	2	0.7	0	0.0	2	1.0
	all	274	-	75	-	199	-

Values are shown as number of participants and %.

**Table 2:** Factor analysis of the 29 items of the BJSQ psychological and physical stress reactions.

	No.	Question	ML1	ML2	ML3	ML4	ML5
Lack of Vigor	Q01	I have been very active		0.86			
	Q02	I have been full of energy		0.88			
	Q03	I have been lively		0.91			
Irritability	Q04	I have felt angry			0.86		
	Q05	I have been inwardly annoyed or aggravated			0.87		
	Q06	I have felt irritable			0.70		
Fatigue	Q07	I have felt extremely tired				0.81	
	Q08	I have felt exhausted				0.84	
	Q09	I have felt weary or listless				0.52	
Anxiety	Q10	I have felt tense	0.36				
	Q11	I have felt worried or insecure	0.74				
	Q12	I have felt restless	0.65				
Depressed Mood	Q13	I have been depressed	0.71				
	Q14	I have thought that doing anything was a hassle	0.42				
	Q15	I have been unable to concentrate	0.57				
	Q16	I have felt gloomy	0.69				
	Q17	I have been unable to handle work	0.62				
	Q18	I have felt sad	0.59				
Somatic Symptoms	Q19	I have felt dizzy					0.34
	Q20	I have experienced joint pains					0.41
	Q21	I have experienced headaches					0.52
	Q22	I have had a stiff neck and/or shoulders					0.60
	Q23	I have had lower back pain					0.54
	Q24	I have had eyestrain					0.50
	Q25	I have experienced heart palpitations or shortness of breath					0.34
	Q26	I have experienced stomach and/or intestine problems					0.32
	Q27	I have lost my appetite	0.32				
	Q28	I have experienced diarrhea and/or constipation					0.32
	Q29	I have not been able to sleep well	0.29				

This table presents the pattern matrix, extracted using the maximum likelihood method (scree plot showing eigenvalue attenuation concluded that a five-factor (ML1–ML5) solution was reasonable); rotation method: Oblimin rotation.

**Table 3:** Intakes of seventeen nutrients in each subgroup.

	Lack of Vigor			Irritability			Fatigue			Somatic Symptoms			Cut-Off
	Low	High	*p*	Low	High	*p*	Low	High	*p*	Low	High	*p*	
K (mg)	2436.8	2538.5	0.007	2531.0	2349.1	0.000	2544.8	2353.8	0.000	2507.0	2441.6	0.129	>2500
Mg (mg)	265.3	278.3	0.002	276.5	256.2	0.000	278.1	256.5	0.000	274.4	265.8	0.064	>270
P (mg)	1071.4	1114.5	0.005	1106.9	1045.1	0.001	1107.4	1054.4	0.001	1100.1	1074.8	0.143	>1100
Fe (mg)	8.07	8.38	0.006	8.36	7.80	0.004	8.34	7.93	0.000	8.22	8.16	0.611	>8.1
Zn (mg)	8.29	8.72	0.014	8.59	8.16	0.006	8.58	8.26	0.035	8.57	8.34	0.775	>8.5
Cu (mg)	1.14	1.18	0.034	1.18	1.10	0.002	1.18	1.11	0.000	1.17	1.14	0.207	>1.18
VA (μgRE)	574.0	564.1	0.003	569.9	570.2	0.010	565.8	577.8	0.000	609.9	520.2	0.053	>570
β-Cx (μg)	221.0	261.7	0.003	261.2	179.9	0.004	274.5	168.8	0.000	253.6	217.4	0.011	>260
β-Ct (μg)	3045.1	3294.1	0.040	3240.8	2914.5	0.005	3272.1	2910.9	0.000	3173.0	3111.3	0.100	>3200
VB1 (mg)	0.96	0.99	0.040	0.99	0.94	0.008	0.99	0.95	0.027	0.99	0.96	0.308	>0.97
Niacin (mgNE)	31.86	33.53	0.001	33.22	30.89	0.000	33.27	31.18	0.000	33.23	31.67	0.025	>33
VB6 (mg)	1.21	1.28	0.003	1.27	1.16	0.000	1.28	1.16	0.000	1.26	1.21	0.057	>1.25
FA (μg)	295.1	316.1	0.001	311.8	283.6	0.000	311.9	288.1	0.000	311.2	294.1	0.026	>300
PA (mg)	5.98	6.22	0.018	6.18	5.82	0.000	6.20	5.84	0.000	6.17	5.96	0.072	>6.0
TDF (g)	18.06	18.90	0.009	18.84	17.32	0.000	18.78	17.68	0.000	18.69	18.03	0.038	>18
IDF (g)	11.63	12.23	0.007	12.24	10.97	0.000	12.19	11.27	0.000	12.06	11.64	0.073	>12
Biotin (μg)	40.60	42.66	0.003	42.07	39.88	0.003	42.23	39.93	0.000	42.48	40.12	0.065	>42

Values are shown as means of each subgroup (low: participants have response score ≤ median; high: participants have response score > median, as for the lack of vigor, the high subgroup has a better psychosomatic status than the low subgroup, and as for irritability, fatigue, and somatic symptoms, the low group has a better status than the high group.). Mann–Whitney U tests were used for comparison between subgroups. VA, vitamin A; μgRE, μg retinol equivalents; β-Cx, beta-cryptoxanthin; β-Ct, beta-carotene; VB1, vitamin B1; mgNE, mg niacin equivalents; VB6, vitamin B6; FA, folic acid; PA, pantothenic acid; DF, dietary fiber. Cut-off: values were set at all four items of MHCs to be above the mean intake of the subpopulation below the median of response score in each item (in lack of vigor, mean intake of subpopulation above the median of response score).

## Data Availability

Data Availability Statement: The data obtained from the “*Sukoyaka* Health Survey” are not currently in a publicly accessible repository but are scheduled to be released in 2024 from a publicly accessible repository managed by the DDBJ (DNA Data Bank of Japan) JGA (Japanese Genotype-phenotype Archive; https://www.ddbj.nig.ac.jp/jga/index-e.html).
